# Complete Genome Sequence of *Mycobacterium bovis* BCG Korea, the Korean Vaccine Strain for Substantial Production

**DOI:** 10.1128/genomeA.00069-13

**Published:** 2013-03-14

**Authors:** Sun Myung Joung, Sung Joo Jeon, Young Ju Lim, Jong-Sung Lim, Beom-Soon Choi, Ik-Young Choi, Jeong Hee Yu, Kyung-In Na, En-Hi Cho, Sang-Sook Shin, Young Kil Park, Chang-Ki Kim, Hee-Jin Kim, Sung Weon Ryoo

**Affiliations:** a Korean Institute of Tuberculosis, Osong, Chungbuk, Republic of Korea; b National Instrumentation Center for Environmental Management, Seoul National University, Seoul, Republic of Korea; c Division of HIV and TB Control, Korea Centers for Disease Control and Prevention, Osong, Chungbuk, Republic of Korea

## Abstract

*Mycobacterium bovis* bacillus Calmette-Guérin (BCG) is the only vaccine available against tuberculosis, and the strains used worldwide represent a family of daughter strains with distinct genotypic characteristics. Here, we report the complete genome sequence of *M. bovis* BCG Korea, the strain that will be actually used in Korea for vaccine production.

## GENOME ANNOUNCEMENT

*Mycobacterium bovis* bacillus Calmette-Guérin (BCG) remains the only vaccine available against tuberculosis (TB). The attenuated strain was derived from an *M. bovis* strain isolated after 230 serial passages on glycerol-potato-bile medium from 1908 to 1921 by Albert Calmette and Camille Guérin at the Institut Pasteur in Lille, France (1–3). It was distributed to laboratories worldwide and maintained in culture until the 1960s, and BCG became widespread after its introduction into the World Health Organization (WHO) Expanded Program on Immunization (EPI) in 1974 (4).

During the Korean War, in 1952, the Korean government began a nationwide BCG vaccination project. In 1954, the Korean National Institution of Prevention & Epidemiology (NIPE), which is now the Korean National Institute of Health (KNIH), began BCG vaccine production, and the mass production of the liquid BCG vaccine (20,000 ml) was initiated in 1960. In 1972, KNIH decided to transfer the main role in BCG production to the Korean Institute of Tuberculosis (KIT), which is affiliated with the Korean National Tuberculosis Association (KNTA). Actual production of a freeze-dried BCG vaccine was started, and its potency proven, by the WHO in 1977. The strain used for production in Korea from 1954 to 2006 was *M. bovis* Pasteur 1173P. The dedication and professionalism of Gilles Marchal at the Institut Pasteur in Paris have been an important contribution in BCG production in Korea and in making the BCG Korea strain.

The original strain of BCG, maintained at the Institut Pasteur in Lille, France, produced hundreds of “daughter” strains and is the progenitor of the most commonly used vaccines. The different “BCG vaccines” produced worldwide comprise a heterogeneous family of daughter strains (5, 6). The complete genome sequence has been determined for only four BCG strains, BCG-Pasteur (7), BCG-Tokyo (8), BCG-Tice (9), and BCG-Moreau (10), and the draft sequences for strains China, Denmark, and Russia (11) have been reported.

We analyzed the whole-genome DNA sequence of *M. bovis* BCG Korea through a shotgun sequencing method using the GS-FLX Titanium system. The sequence data were assembled with Newbler software (version 2.5.3; Roche, Germany). A total of 73 contigs were produced in 10 scaffolds through *de novo* assembly. Gaps among the contigs were closed by primer walking on standard PCR products. We corrected the homopolymeric region error of the consensus sequence using the Illumina HiSeq system. Coding genes and pseudogenes across the genome were predicted using Glimmer (12) and were annotated by comparison with the NCBI nonredundant (NR) database (13). The annotation results were verified using Artemis (14).

The *M. bovis* BCG Korea genome is one chromosome of 4,376,711 bp with a G+C content of 65.64% and 4,142 coding sequences (CDS). *M. bovis* BCG Korea has one rRNA operon and 45 tRNA genes (15, 16).

### Nucleotide sequence accession number.

The complete genome sequence of *M. bovis* BCG Korea has been assigned the GenBank accession number CP003900. 
